# Exosome long non-coding RNA SOX2-OT contributes to ovarian cancer malignant progression by miR-181b-5p/SCD1 signaling

**DOI:** 10.18632/aging.203645

**Published:** 2021-10-24

**Authors:** Yongjing Lai, Lihua Dong, Huifang Jin, Hongju Li, Meiling Sun, Jianlan Li

**Affiliations:** 1 Department of Obstetrics, People’s Hospital of Rizhao, Rizhao, China; 2 Department of Health Emergency Office, Rizhao Center for Disease Control and Prevention, Rizhao, China; 3 Department of Gynaecolgy, People’s Hospital of Rizhao, Rizhao, China

**Keywords:** ovarian cancer, progression, SOX2-OT, exosome, miR-181b-5p

## Abstract

Ovarian cancer is a common gynecologic cancer with increased mortality and morbidity. Exosome-delivered long non-coding RNAs have been well found in cancer development. However, the function of exosomal SOX2-OT in ovarian cancer development is still unreported. In the present study, we were interested in the investigation of the effect of exosomal SOX2-OT during ovarian cancer pathogenesis. Significantly, we revealed that the SOX2-OT expression levels were up-regulated in the ovarian cancer patients’ plasma exosomes. The depletion of exosomal SOX2-OT inhibited migration, invasion, and proliferation and induced apoptosis in ovarian cancer cells. In mechanical exploration, SOX2-OT could sponge miR-181b-5p, and miR-181b-5p was able to target SCD1 in the ovarian cancer cells. The SCD1 overexpression and miR-181b-5p inhibitor could reverse exosomal SOX2-OT-mediated ovarian cancer progression. Functionally, the depletion of exosomal SOX2-OT significantly reduced tumor growth of ovarian cancer cells *in vivo*. In summary, we concluded that exosomal SOX2-OT enhanced ovarian cancer malignant phenotypes by miR-181b-5p/SCD1 axis. Our finding presents novel insights into the mechanism by which exosomal lncRNA SOX2-OT promotes ovarian cancer progression. SOX2-OT, miR-181b-5p, and SCD1 may serve as potential targets for the treatment of ovarian cancer.

## INTRODUCTION

Ovarian cancer serves as the primary reason for women cancer-related mortality in all gynecologic tumors [1]. Despite therapies have been advanced, treatments are limited because of drug-resistance [2]. The diagnosis normally occurs at the more advanced stage in ovarian cancer patients [3]. Current treatment is not available to cure ovarian cancer, and 5-year survival incidence maintains at 45% [4]. Accordingly, understanding the molecular mechanism of ovarian cancer progression will be helpful for the improvement of therapeutic strategies for ovarian cancer [5–7]. However, the investigation of the molecular mechanism of ovarian cancer development is still limited.

The 30-100 nm nano-sided particles termed exosomes are extracellularly delivered after multivesicular endosome fusion [8, 9]. The exosomes transfer long non-coding RNAs, microRNA (miRNAs), (lncRNAs), and some active materials, playing crucial roles in the modulation of cancer pathogenesis [10, 11]. Exosomes-derived non-coding RNAs have been identified to participate in ovarian cancer progression [12, 13]. Meanwhile, lncRNA SOX2-OT is able to load in the exosome and is involved in the regulation of multiple cancers, including prostate cancer, nasopharyngeal carcinoma, and lung squamous cell carcinoma [14–16]. Meanwhile, it has been identified the clinical significance of lncRNA SOX2-OT in ovarian cancer cells and clinical tissues [17]. SOX2-OT contributes to motility and proliferation of ovarian cancer cells (Han, 2018 #25). However, the function of exosomal SOX2-OT during ovarian cancer development is still unreported. Hence, the exploration of the exosomal SOX2-OT effect on the ovarian cancer progression is innovative.

MicroRNAs (miRNAs) presents fundamental roles in many cellular mechanisms *via* regulating numerous genes at the post-transcriptional level [18, 19]. Several miRNAs are involved in the tumorigenesis of ovarian cancer. For instance, MiR-126-3p decreases invasion and proliferation by inhibiting PLXNB2 in ovarian cancer [20]. MiR-34a inhibits chemoresistance and cell proliferation of ovarian cancer by regulating HDAC1 [21]. Meanwhile, miR-181b-5p serves as a tumor suppressor for multiple cancers, such as prostate cancer, non-small cell lung cancer, and colorectal cancer [22–24]. Moreover, sterol CoA desaturase (SCD1), a lipid regulating enzyme, is up-regulated in cancer cells to facilitate malignancies, containing liver cancer, lung cancer, breast cancer [25–27]. Besides, it has been found that SCD1 is able to regulate ferroptosis in ovarian cancer [28]. Nevertheless, the effect of exosomal SOX2-OT on miR-181b-5p and SCD1 during ovarian cancer pathogenesis remains unclear. Consequently, it is innovative to investigate the function of exosomal SOX2-OT/miR-181b-5p/SCD1 signaling in ovarian cancer development.

In this study, we focused on the exploration of exosomal SOX2-OT function in ovarian cancer. We uncovered the new role of exosomal SOX2-OT in enhancing ovarian cancer malignant phenotype by modulating miR-181b-5p/SCD1 axis.

## RESULTS

### The expression of SOX2-OT is enhanced in the plasma exosome of ovarian cancer patients

**Figure 1:**
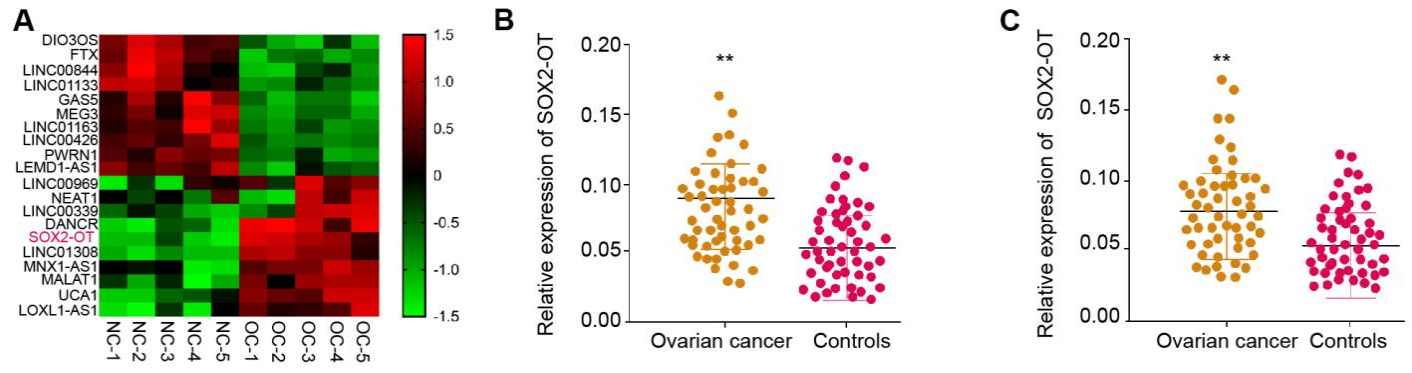
**The expression of SOX2-OT is enhanced in the plasma exosome of ovarian cancer patients.** (**A**) The lncRNA profiling was performed in the exosomes from ovarian cancer patients (n = 5) and normal controls (n = 5). (**B**) The expression of SOX2-OT was tested by qPCR in the plasma exosome from ovarian cancer patients (n = 55). (**C**) The expression of SOX2-OT was analyzed by qPCR in the ovarian cancer patients (n = 55) and the adjacent normal tissues (n = 55).

Firstly, we performed the lncRNA profiling in the exosomes from ovarian cancer patients (n = 5) and normal samples (n = 5) and identified the elevated expression of SOX2-OT in the exosomes from ovarian cancer patients compared with normal samples (Figure 1A). To assess the potential correlation of exosomal SOX2-OT with the ovarian cancer progression, we analyzed their expression in the plasma exosome of ovarian cancer patients. Significantly, the expression of SOX2-OT was enhanced in the plasma exosome from ovarian cancer patients (n = 55) relative to the normal cases (n = 55) (Figure 1B). In addition, ovarian cancer patient tissues (n = 55) presented higher expression of SOX2-OT compared with the adjacent normal tissues (n = 55) (Figure 1C). Furthermore, TEM analysis revealed that exosomes from the ovarian cancer patients showed the same size as the normal cases (Supplementary Figure 1A). Similarly, the existence of the exosome markers, such as TSG101 and CD63, in the exosome of ovarian cancer patients and normal cases, was validated (Supplementary Figure 1B).

### Exosomal SOX2-OT increases proliferation and attenuates apoptosis of ovarian cancer cells

**Figure 2:**
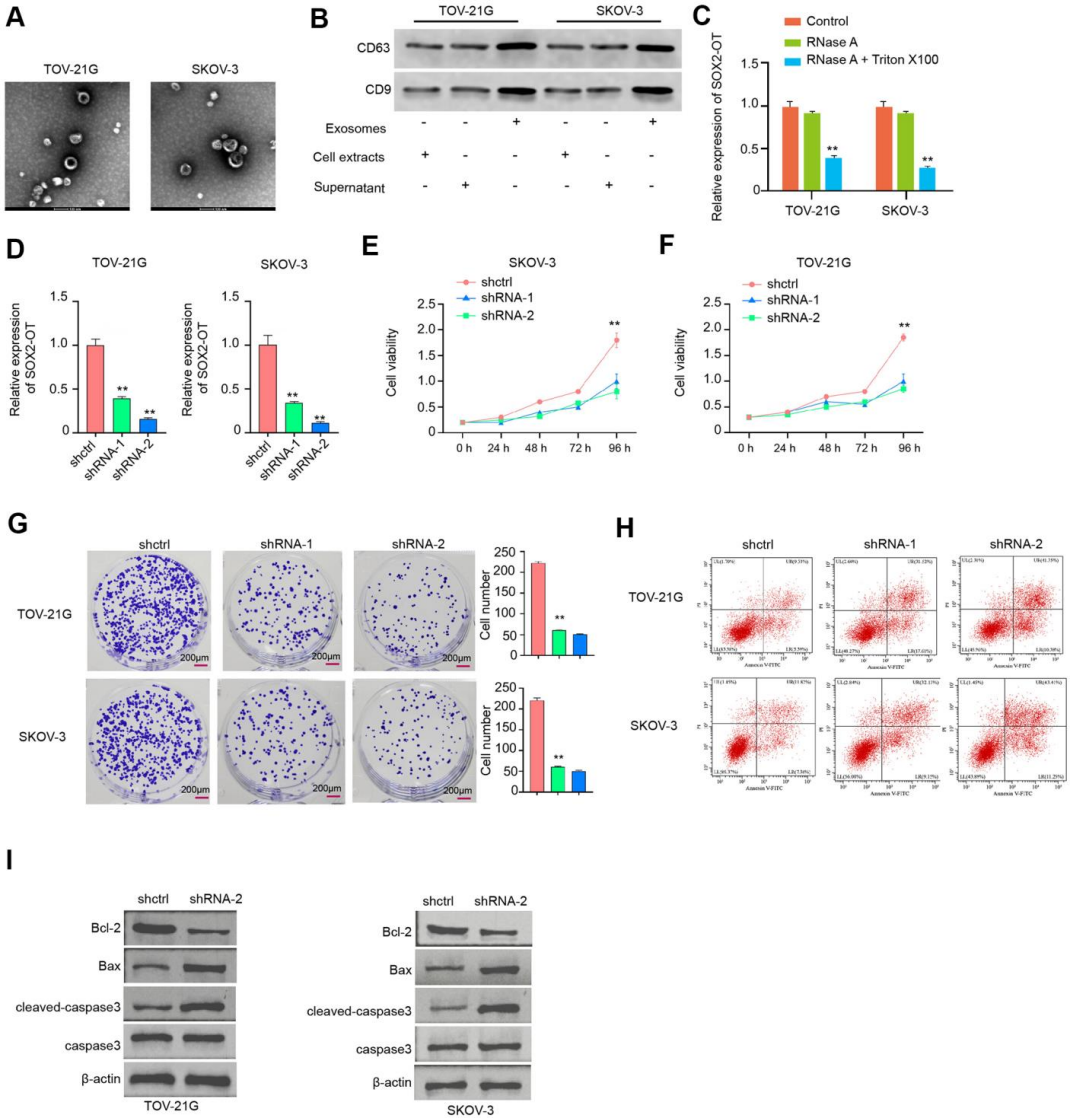
**Exosomal SOX2-OT increases proliferation and attenuates apoptosis of ovarian cancer cells.** (**A**) The characteristics of exosomes were measured by the TEM in the TOV-21G and SKOV-3 cells. (**B**) The expression of CD9 and CD63 was tested by the Western blot analysis in the exosome of TOV-21G and SKOV-3 cells. (**C**) The expression of SOX2-OT was analyzed by qPCR in the TOV-21G and SKOV-3 cells treated with RNase A (1 μg/mL) or co-treated with RNase A (1 μg/mL) and Triton X100 (0.1%). (**D**–**I**) The TOV-21G and SKOV-3 cells were treated with the SOX2-OT shRNA or control shRNA, and the exosomes were extracted and further treated the cells. (**D**) The efficiency of the SOX2-OT knockdown was confirmed by qPCR assays in the cells. (**E**, **F**) The cell viability was assessed by the MTT assays in the cells. (**G**) The cell proliferation was determined by colony formation assays in the cells. (**H**) The cell apoptosis was examined by flow cytometry analysis in the cells. (**I**) The expression of Bcl-2, Bax, cleaved-caspase3, and caspase3 was measured by Western blot analysis in the cell. Data are presented as mean ± SD. Statistic significant differences were indicated: * *P* < 0.05, ** *P* < 0.01, *** *P* < 0.001.

Next, the role of exosomal SOX2-OT in ovarian cancer was analyzed. The exosome was extracted from the culture medium of TOV-21G and SKOV-3 cells and the characteristic were shown by TEM (Figure 2A). Besides, the expression levels of CD63 and CD9 were identified in the exosome of the TOV-21G and SKOV-3 cells (Figure 2B). In addition, the SOX2-OT expression was tested in culture medium treated with RNase A or co-treated with RNase A and Triton X-100. Our data showed that the SOX2-OT expression was unacted on the treatment of RNase A while significantly reduced upon the simultaneous co-treatment of RNase A and Triton X-100 (Figure 2C). Next, the efficiency of SOX2-OT depletion was validated in the TOV-21G and SKOV-3 cells (Figure 2D). Significantly, the cell proliferation was reduced by the depletion of SOX2-OT in the cells (Figure 2E). Meanwhile, the colony formation assays confirmed the similar results in the cells (Figure 2F). Moreover, the SOX2-OT knockdown induced the apoptosis of TOV-21G and SKOV-3 cells (Figure 2G, 2H). Consistently, the depletion of SOX2-OT reduced Bcl-2 expression while enhanced Bax and cleaved-caspase3 expression in the cells (Figure 2I).

### Exosomal SOX2-OT induces invasion and migration

**Figure 3:**
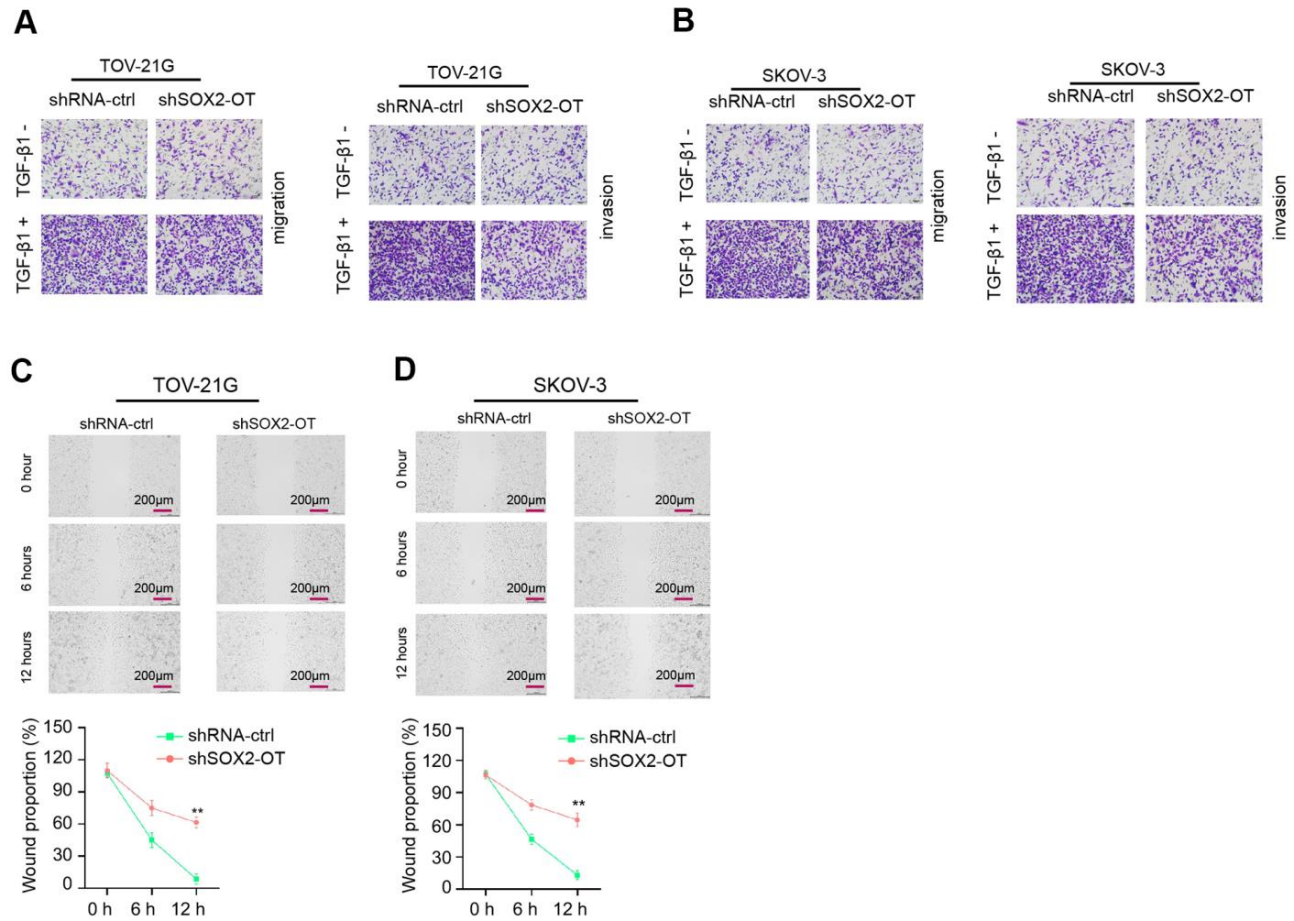
**Exosomal SOX2-OT induces invasion and migration of ovarian cancer cells.** (**A**–**D**) The TOV-21G and SKOV-3 cells were treated with control shRNA or SOX2-OT shRNA, and the exosomes were extracted and further treated the cells. (**A**, **B**) The cells were treated with or without TGF-β1 (5 ng/ml). The cell migration and invasion were analyzed by transwell assays in the cells. (**C**, **D**) The migration and invasion were determined by wound healing assays in the cells. The wound healing proportion was shown. Data are presented as mean ± SD. Statistic significant differences were indicated: * *P* < 0.05, ** *P* < 0.01.

Then, we revealed that TOV-21G and SKOV-3 cell invasion and migration were increased by SOX2-OT knockdown (Figure 3A, 3B). Similarly, the depletion of SOX2-OT significantly enhanced wound healing proportion (Figure 3C, 3D), indicating that exosomal SOX2-OT induces invasion and migration.

### SOX2-OT serves as a miR-181b-5p sponge in ovarian cancer cells

**Figure 4:**
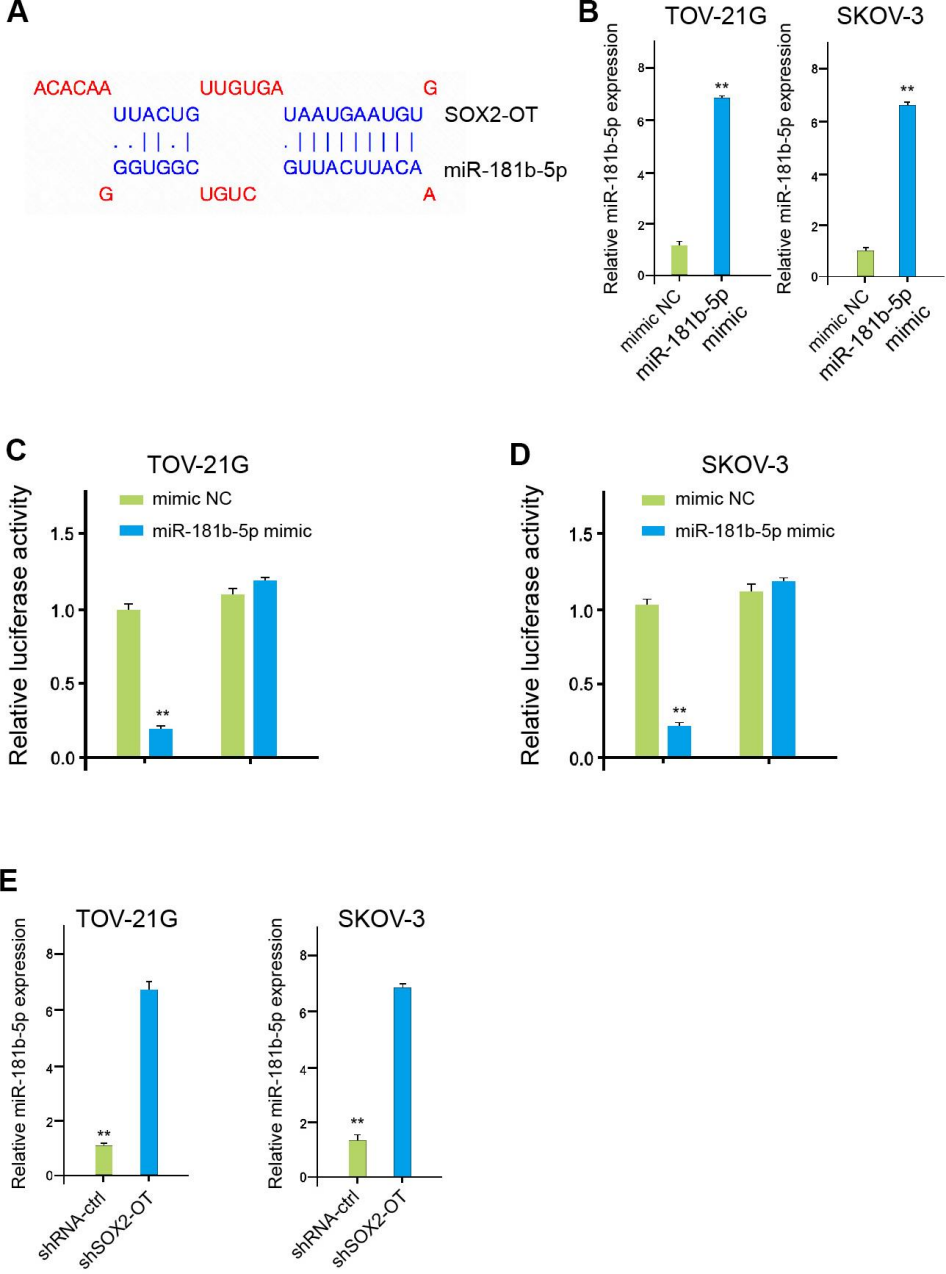
**SOX2-OT serves as a miR-181b-5p sponge in ovarian cancer cells.** (**A**) The interaction of SOX2-OT and miR-181b-5p was analyzed by bioinformatic analysis based on ENCORI (http://starbase.sysu.edu.cn/index.php). (**B**) The expression levels of miR-181b-5p were assessed by qPCR in the TOV-21G and SKOV-3 cells treated with control mimic or miR-181b-5p mimics. (**C**, **D**) Luciferase activities of SOX2-OT (SOX2-OT WT) and SOX2-OT with the miR-181b-5p-binding site mutant (SOX2-OT MUT) were determined by luciferase reporter gene assays in the TOV-21G and SKOV-3 cells treated with control mimic or miR-181b-5p mimic. (**E**) The TOV-21G and SKOV-3 cells were treated with control shRNA or SOX2-OT shRNA. The expression of miR-181b-5p was measured by qPCR assays in the cells. Data are presented as mean ± SD. Statistic significant differences were indicated: ** *P* < 0.01.

The binding region between SOX2-OT and miR-181b-5p was predicted in the bioinformatic analysis (Figure 4A). The effectiveness of miR-181b-5p mimic was confirmed in TOV-21G and SKOV-3 cells (Figure 4B). The luciferase activities of SOX2-OT, but not SOX2-OT mutant, were decreased by miR-181b-5p mimic in the cells (Figure 4C, 4D). Consistently, SOX2-OT knockdown down-regulated the miR-181b-5p expression in the TOV-21G and SKOV-3 cells, suggesting that SOX2-OT serves as a miR-181b-5p sponge in ovarian cancer cells (Figure 4E).

### MiR-181b-5p targets SCD1 in ovarian cancer cells

**Figure 5:**
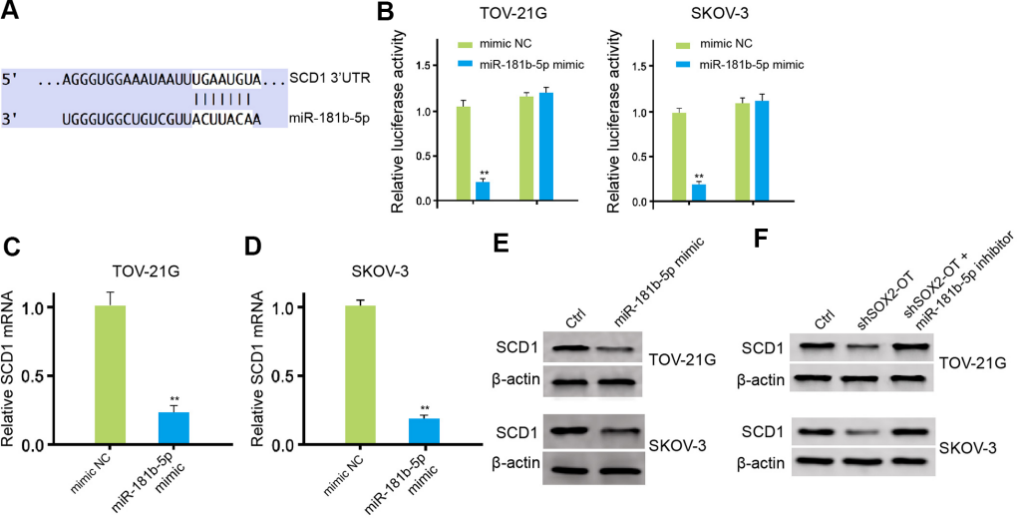
**MiR-181b-5p targets SCD1 in ovarian cancer cells.** (**A**) The binding of miR-181b-5p and SCD1 3’ UTR was analyzed by bioinformatic analysis based on Targetscan (http://www.targetscan.org/vert_72/). (**B**–**D**) The TOV-21G and SKOV-3 cells were treated with control mimic or miR-181b-5p mimic. (B) The luciferase activities of wild type SCD1 (SCD1 WT) and SCD1 with the miR-181b-5p-binding site mutant (SCD1 MUT) were determined by luciferase reporter gene assays in the cells. (**C**, **D**) The mRNA expression of SCD1 was tested by qPCR assays in the cells. (**E**, **F**) The protein expression of SCD1 was measured by Western blot analysis in the cells. The TOV-21G and SKOV-3 cells were treated with control shRNA or SOX2-OT shRNA, co-treated with SOX2-OT shRNA and miR-181b-5p inhibitor. The protein expression of SCD1 was analyzed by Western blot analysis in the cells. Data are presented as mean ± SD. Statistic significant differences were indicated: * *P* < 0.05, ** *P* < 0.01.

The miR-181b-5p-targeted site in SCD1 3’ UTR was found in a bioinformatic analysis (Figure 5A). Significantly, the luciferase activities of SCD1, but not SCD1 mutant, were reduced by miR-181b-5p mimic in the TOV-21G and SKOV-3 cells (Figure 5B). Moreover, the expression level of SCD1 was remarkably down-regulated by miR-181b-5p mimic in the cells (Figure 5C, 5D). In addition, the levels of SCD1 was up-regulated by SOX2-OT knockdown, while the miR-181b-5p inhibitor could reverse this effect in the TOV-21G and SKOV-3 cells (Figure 5E, 5F).

### Exosomal SOX2-OT contributes to the progression of ovarian cancer by miR-181b-5p/SCD1 axis *in vitro*

**Figure 6:**
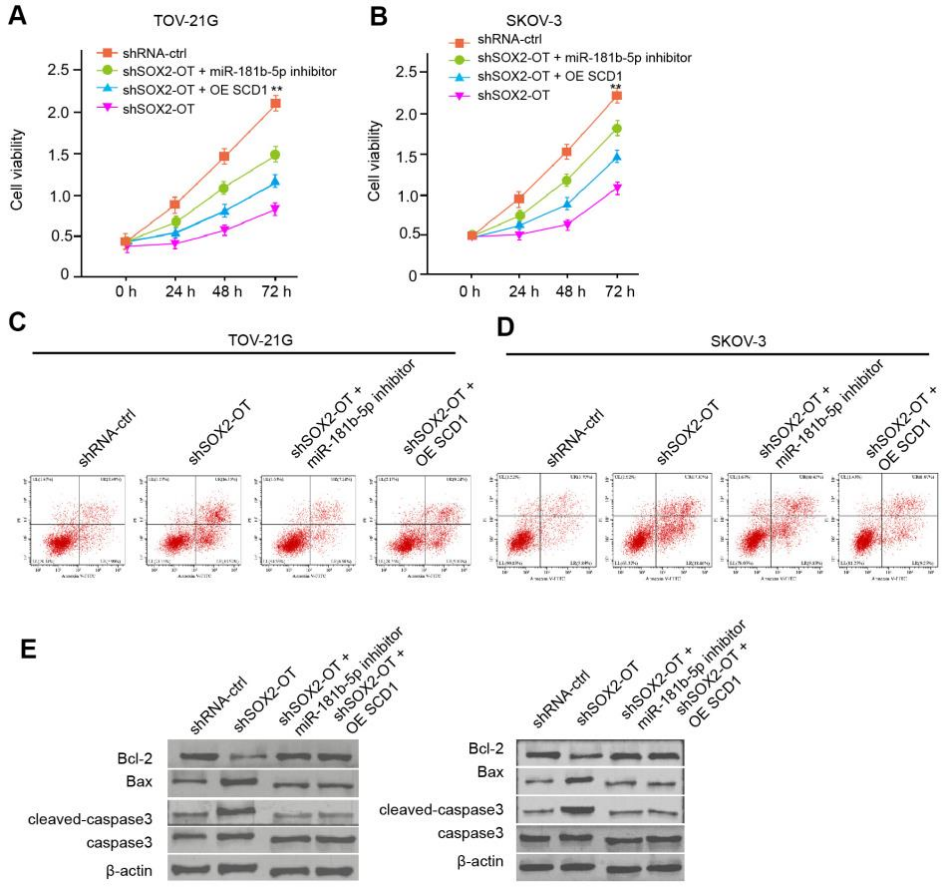
**Exosomal SOX2-OT contributes to the progression of ovarian cancer by miR-181b-5p/ SCD1 axis *in vitro*.** (**A**–**E**) The TOV-21G and SKOV-3 cells were treated with control shRNA or SOX2-OT shRNA, co-treated with SOX2-OT shRNA and miR-181b-5p inhibitor or pcDNA3.1-SCD1. (**A**, **B**) The cell viability was measured by MTT assays in the cells. (**C**, **D**) The cell apoptosis was analyzed by flow cytometry analysis in the cells. (**E**) The expression of Bcl-2, Bax, cleaved-caspase3, and caspase3 was measured by Western blot analysis in the cell. Data are presented as mean ± SD. Statistic significant differences were indicated: ** *P* < 0.01.

Next, we investigated the effect of the exosomal SOX2-OT/miR-181b-5p/SCD1 axis in ovarian cancer progression. The exosomal depletion of SOX2-OT reduced the viability of TOV-21G and SKOV-3 cells, and the miR-181b-5p inhibitor or SCD1 overexpression rescued the phenotype (Figure 6A, 6B). Moreover, the miR-181b-5p inhibitor or SCD1 overexpression was able to reverse the SOX2-OT-depletion-induced apoptosis of TOV-21G and SKOV-3 cells (Figure 6C, 6D). Consistently, the depletion of SOX2-OT reduced Bcl-2 expression while enhanced Bax and cleaved-caspase3 expression in the cells, in which miR-181b-5p inhibitor or SCD1 reversed the effect (Figure 6E).

### Exosomal SOX2-OT promotes tumor growth of ovarian cancer *in vivo*

**Figure 7:**
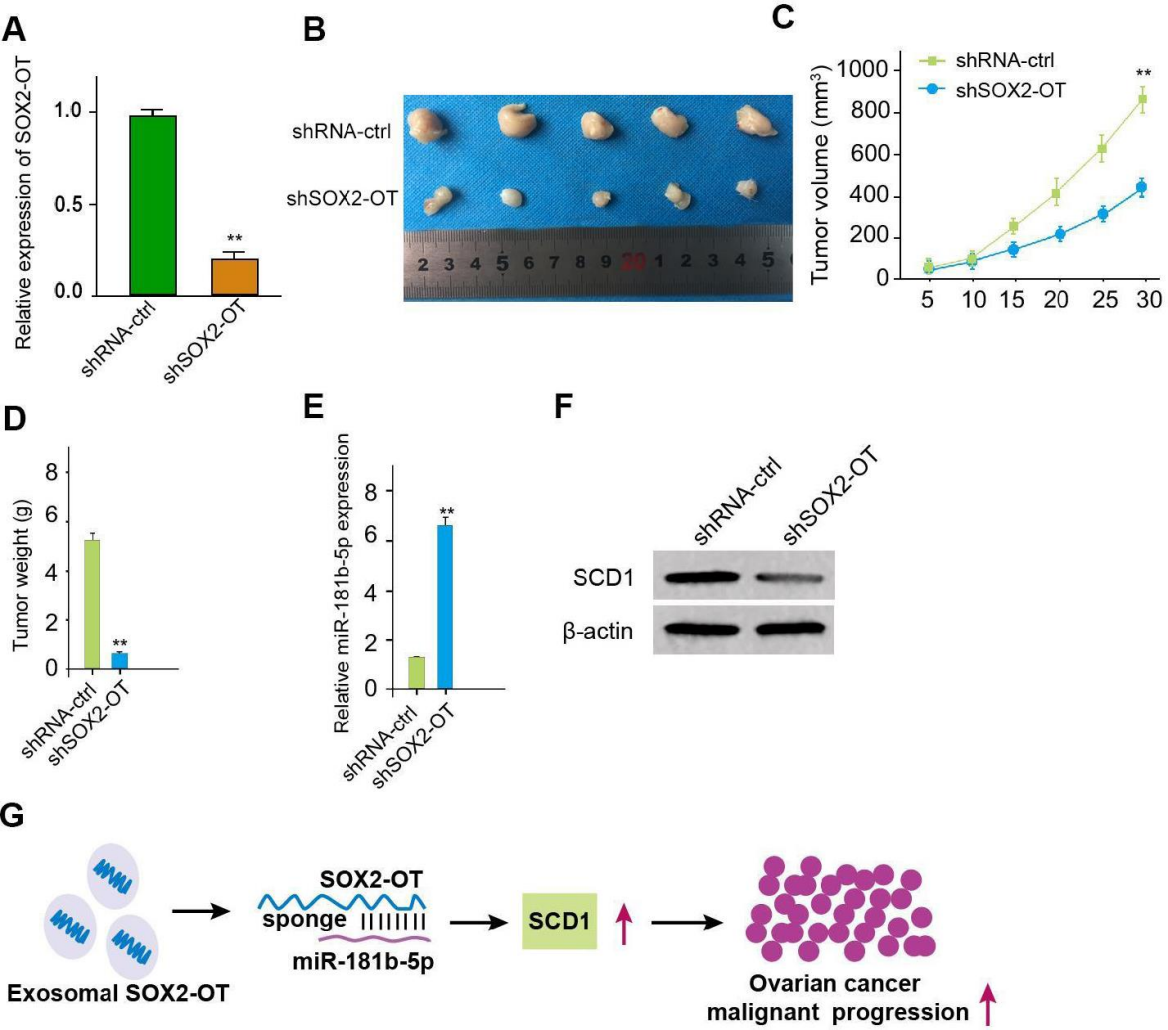
**Exosomal SOX2-OT promotes tumor growth of ovarian cancer *in vivo*.** (**A**–**F**) The impact of exosomal SOX2-OT on tumor growth of ovarian cancer cells *in vivo* was analyzed by nude mice tumorigenicity assay (n = 5). The SKOV-3 cells were treated with control shRNA or SOX2-OT shRNA exosome. (**A**) The expression of SOX2-OT was tested by qPCR in the tumor tissues of the mice. (**B**) Representative images of dissected tumors from nude mice were shown. (**C**) The average tumor volume was calculated and presented. (**D**) The average tumor weight was calculated and presented. (**E**) The expression of miR-181b-5p was measured by qPCR in the tumor tissues of the mice. (**F**) The expression of SCD1 was tested by Western blot analysis in the tumor tissues of the mice. (**G**) The schematic model of the study. Data are presented as mean ± SD. Statistic significant differences were indicated: * *P* < 0.05, ** *P* < 0.01.

We then evaluated the role of exosomal SOX2-OT in the ovarian cancer progression *in vivo* by the tumorigenicity analysis. The expression of inhibited SOX2-OT was validated in the system (Figure 7A). Significantly, the exosomal SOX2-OT depletion could attenuate the tumor size, volume, and weight (Figure 7B–7D). MiR-181b-5p expression was increased but the SCD1 expression down-regulated in tumor tissues (Figure 7E, 7F), indicating that exosomal SOX2-OT promotes tumor growth of ovarian cancer *in vivo*.

## DISCUSSION

Ovarian cancer is the predominant type of gynecologic cancer with severe morbidity and high mortality [1]. Exosomes-derived lncRNAs play critical roles in ovarian cancer [13]. Nevertheless, the function of exosomal SOX2-OT in ovarian cancer development. In the present investigation, we found that exosomal SOX2-OT promoted ovarian cancer malignant phenotype by modulating miR-181b-5p/SCD1 axis.

It has been found that several exosomal non-coding RNAs are involved in the regulation of ovarian cancer. Exosome-derived miR-205 enhances metastasis of ovarian cancer cells by promoting angiogenesis [29]. Circular RNA WHSC1 contributes to progression of ovarian cancer by modulating hTERT and MUC1 through targeting exosomal miR-1182 and miR-145 [30]. Exosomal miR21 promotes paclitaxel resistance by targeting APAF1 in ovarian cancer [31]. The epithelial ovarian cancer exosomal lncRNAs suppress migration of ovarian cancer [32]. In this study, we showed that SOX2-OT was up-regulated in the plasma exosome of ovarian cancer patients. Exosomal SOX2-OT enhanced migration, invasion, and proliferation, and repressed apoptosis of ovarian cancer cells. These data indicate an unreported role of exosomal SOX2-OT in ovarian cancer, indicating informative evidence of crucial roles of exosomal lncRNAs in ovarian cancer.

Moreover, lncRNA normally exert their activity by interacting with miRNAs and various miRNAs contribute to the modulation of ovarian cancer. It has been reported that miR-205 enhances invasion of ovarian cancer cells by inhibiting TCF21 [33]. MiR-34 represses cell proliferation by facilitating apoptosis and autophagy and suppresses cell invasion by regulating Notch1 in ovarian cancer [34]. MiR-204-5p attenuates cell growth by decreasing USP47 expression in ovarian cancer [35]. Furthermore, it has been identified that SCD1 is able to regulate tumorigenesis. SCD1 elevates migration of the triple-negative breast cancer cells by regulating PLD/mTOR axis [36]. SCD1 enhances metastasis by inhibiting PTEN in glucose response of colorectal cancer [37]. SCD1 controls the stemness process by targeting YAP/TAZ signaling in lung cancer [38]. It also has been found that SCD1 preserves ovarian cancer cells against ferroptosis [28]. Our mechanism research revealed that SOX2-OT sponged miR-181b-5p and miR-181b-5p targeted SCD1 in ovarian cancer cells. The SCD1 overexpression and miR-181b-5p inhibitor could reverse exosomal SOX2-OT-mediated ovarian cancer progression. It uncovers a novel function of SOX2-OT/miR-181b-5p/SCD1 axis in ovarian cancer development, presenting the unreported mechanism involving SOX2-OT, miR-181b-5p, and SCD1.

In conclusion, we identified that exosomal SOX2-OT contributed to ovarian cancer malignant phenotype by miR-181b-5p/SCD1 axis (Figure 7G). Our finding describes novel insights in the mechanism that exosomal lncRNA SOX2-OT promotes ovarian cancer progression. SOX2-OT, miR-181b-5p and SCD1 may be utilized as therapeutic targets.

## MATERIALS AND METHODS

### Ovarian cancer clinical samples

The 55 ovarian cancer clinical samples were collected from People’s Hospital of Rizhao. All the cases were detected by histopathological measurement and performed the diagnosis by two clinicians. Samples collected from the patients and stored at -80° C. The lncRNA profiling was performed (Annoroad, China). The samples applied in this research were under the written approval of the cases. All experiments are consistent with guidelines of Ethics Committee of People’s Hospital of Rizhao.

### Exosome isolation and analysis

The Exosomes were isolated and verified as the previous report [39]. The plasma and culture medium were centrifuged for 15 minutes at the condition of 3000 × g to eliminate cell debris. Then, the exosomes were extracted by an Exoquick exosome precipitation solution (System Biosciences, USA). The exosomes were observed and validated using the transmission electron microscopy (TEM, Hitachi, Japan).

### Cell culture and treatment

The TOV-21G and SKOV-3 cells were maintained in the lab. The TOV-21G and SKOV-3 cells were incubated at the incubator of 5% CO_2_ and 37° C in the DMEM medium (Hyclone, USA) with penicillin (100 units/mL, Hyclone, USA), streptomycin (0.1 mg/mL, Hyclone, USA), and FBS (10%, Hyclone, USA). The control shRNA, SOX2-OT shRNA, pcDNA3.1-SCD1, mimic and inhibitor of miR-181b-5p were purchased from GenePharma (China). SOX2-OT shRNA-1: GCACCGCTATACAGAGAAACCTTATCCTCGAGGATAAGGTTTCTCTGTATAGCTTTTTTG; SOX2-OT shRNA-2: GCACCGGAGCAAAGGTGCTGTCATTTCTCGAGAAATGACAGCACCTTTGCTCCTTTTTG.

### Quantitative reverse transcription-PCR (qRT-PCR)

Cells were collected after indicated treatment, and total RNA was extracted using TRIZOL (Invitrogen, USA), and reversely transcribed to cDNA using a Reverse Transcription System Ki (TaKaRa, China). The expression was determined by a SYBR Mix kit (Takara, China) in a Real-time PCR system (BD Biosciences, USA) and analyzed using 2^-ΔΔCt^ method. Primer sequences: SOX2-OT F: 5′-GTTCATGGCCTGGACTCTCC-3′, R: 5′-ATTGCTAGCCCTCACACCTC-3′; miR-181b-5p F: 5′-CTCAACTGGTGTCGTGGAGTCGG-3′, R: 5′-CAATTCAGTTGAGTTGCATTC-3′; SCD1 F: 5′- AAACCTGGCTTGCTGATG-3′, R: 5′-GGGGGCTAATGTTCTTGTCA-3′; GAPDH F: 5′-AAGAAGGTGGTGAAGCAGGC-3′, R: 5′-TCCACCACCCAGTTGCTGTA-3′; U6 F: 5′-GCTTCGGCAGCACATATACTAA-3′, R: 5′-AACGCTTCACGAATTTGCGT-3′.

### MTT assay

The cell viability of TOV-21G and SKOV-3 cells was assessed by a 3-(4, 5- dimethylthiazol-2-yl)-2,5-diphenyltetrazolium bromide (MTT) experiment. TOV-21G and SKOV-3 cells were treated as indicated in each experiment, digested, suspended as single cells, and planted in 96-well plates (3×103 cells per well). MTT reagent were added at a final concentration of 5 mg/ml in each well. Following a 4-hours incubation, the cell medium was discarded and replaced by 100 μl DMSO in each well. The plates were gently shaken in dark for 10 minutes. Absorbance at 490nm was detected.

### Colony formation assays

TOV-21G and SKOV-3 cells were planted into 6-well plates with 1000 cells in each well after appropriate transfection, and cultured in 37° C incubator for two weeks. After the visible colonies formed, cells were stained with crystal violet diluted in methanol for 20 minutes. The colonies were captured and counted via a microscope (Olympus, Japan).

### Transwell assays

The migration and invasion ability of TOV-21G and SKOV-3 cells were determined via using a transwell chamber (Corning, USA). The cells were treated with or without TGF-β1 (5 ng/ml). To detect migration, TOV-21G and SKOV-3 (2 × 104 cells/well) were seeded into the upper chambers with FBS-free medium, while the lower chambers were filled with complete DMEM medium. After 24 hours incubation, the membranes of upper chambers were fixed by 4% paraformaldehyde for 15 min and stained by 0.5% crystal violet for 30 minutes. The migrated cells were photographed and counted. For cell invasion, the process was similar with that of migration experiment, only that the upper chambers were coated with Matrigel (BD Bioscience, USA).

### Wound healing assay

TOV-21G and SKOV-3 cells were planted in 4-well plate at a density of 3×105/well to form a monoplayer. A sterilized 200 ul pipette were used to gently scratch a line on the monoplayer. Then the cells were washed with PBS to wash out the detached cells, and replaced with fresh FBS-free medium for continuing incubation. The images of scratch were captured at 0 h, 6 h and 12 h after scratching with a microscope (Olympus), and measured.

### Analysis of cell apoptosis

The transfected TOV-21G and SKOV-3 cells were digested and washed, suspended in 100 μL binding buffer and stained with Annexin V-FITC and PI staining reagents under the instruction of detection kit (Keygen, China) n dark condition for 20 minutes. Afterward, the cells were washed and resuspended in binding buffer and measured by a flow cytometry (BD Biosciences, USA) immediately.

### Luciferase reporter gene assay

The pmirGLO-SCD1, pmirGLO-SCD1 mutant or pmirGLO-SOX2-OT, pmirGLO-SOX2-OT mutant, and miR-181b-5p mimic or control mimic were synthesized and obtained (GenePharma, China). Briefly, the cells were transfected with the pmirGLO-SCD1, pmirGLO-SCD1 mutant or pmirGLO-SOX2-OT, pmirGLO-SOX2-OT mutant, and miR-181b-5p mimic or control mimic. Renilla was applied as a normalized control.

### Western blot analysis

FTC-133 and TPC-1 cells received indicated treatment were washed in PBS and lysed with ice-cold lysis buffer containing a cocktail of proteinase inhibitors. Total protein was divided in SDS-PAGE gel and shifted to NC membranes. The membranes were soaked in fast blocking buffer for 15 minutes and incubated with specific primary antibodies, namely the TSG101 (Abcam, USA), CD63 (Abcam, USA), Grp94 (Abcam, USA), CD9 (Abcam, USA), SCD1 (Abcam, USA), Bcl-2 (Abcam, USA), Bax (Abcam, USA), cleaved-caspase3 (Abcam, USA), caspase3 (Abcam, USA), and β-actin (Abcam, USA), at 4° C overnight. Next day, the membranes were incubated with corresponding HRP-conjugated secondary antibodies and ECL substrate (Beyotime, China). The visualization of proteins was performed by a Gel imaging system (BD Biosciences, USA).

### Analysis of tumorigenicity in nude mice

All animal experiments in this work were authorized by Ethics Committee of People’s Hospital of Rizhao. BALB/c nude mice aged 4-week were maintained in a specific pathogen-free (SPF) environment. The mice were randomly divided into two groups (n=5). A total number of 1 × 107 SKOV-3 cells transfected with control shRNA or SOX2-OT shRNA exosome were collected, washed, suspended in 100 μL saline, and then subcutaneously injected into the left fat pad of each mice. The tumor size (volume = 0.5×width^2^ × length) and body weight were measured at the indicated time points.

### Statistical analysis

Data in this study were repeated at least three times, shown as means ± SD and analyzed by a GraphPad prism 7 software. The statistical significance were defined by p < 0.05 in Student’s t test or one-way ANNOVA analysis.

## Supplementary Material

Supplementary Figure 1
