# Supplementary material for: Exosome long non-coding RNA SOX2-OT contributes to ovarian cancer malignant progression by miR-181b-5p/SCD1 signaling
Source: Aging (Albany NY). 2021 Oct 24;13(20):23726–38. doi: 10.18632/aging.203645 (PMC8580347; doi:10.18632/aging.203645)
Supplement: Supplementary Figure 1 [file aging-13-203645-s001.pdf]

SUPPLEMENTARY FIGURE

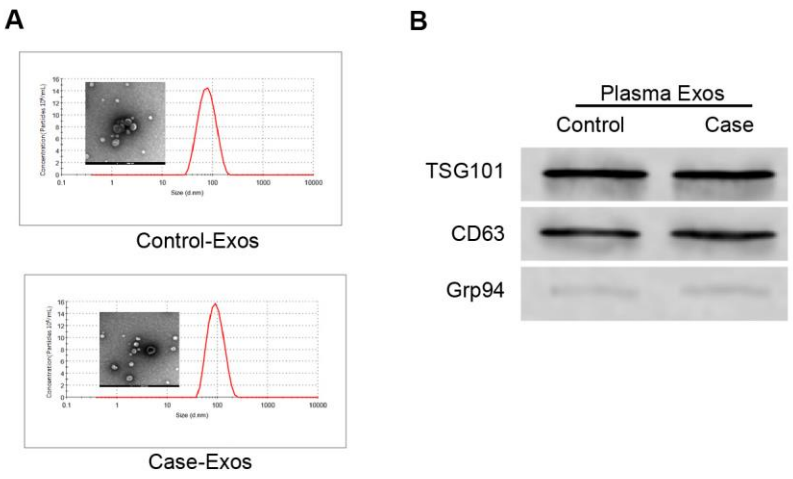

Supplementary Figure 1. The identification of plasma exosome from ovarian cancer patients. (**A**) The characteristics of exosomes were assessed by the TEM in the ovarian cancer patients. (**B**) The expression of TSG101, CD63, and Grp94 was measured by Western blot analysis in the exosome from ovarian cancer patients.
